# A Digital Library for Increasing Awareness About Living Donor Kidney Transplants: Formative Study

**DOI:** 10.2196/17441

**Published:** 2020-07-21

**Authors:** Amy D Waterman, Emily H Wood, Omesh N Ranasinghe, Amanda Faye Lipsey, Crystal Anderson, Wendy Balliet, Lauren Holland-Carter, Stacey Maurer, Maria Aurora Posadas Salas

**Affiliations:** 1 Division of Nephrology David Geffen School of Medicine University of California, Los Angeles Los Angeles, CA United States; 2 Terasaki Research Institute Los Angeles, CA United States; 3 Medical University of South Carolina Charleston, SC United States

**Keywords:** living donor kidney transplant, living donation, health education, informed decision-making, awareness, health literacy, video library, health technology, kidney diseases, diffusion of innovation, digital library, mobile phone

## Abstract

**Background:**

It is not common for people to come across a living kidney donor, let alone consider whether they would ever donate a kidney themselves while they are alive. Narrative storytelling, the sharing of first-person narratives based on lived experience, may be an important way to improve education about living donor kidney transplants (LDKTs). Developing ways to easily standardize and disseminate diverse living donor stories using digital technology could inspire more people to consider becoming living donors and reduce the kidney shortage nationally.

**Objective:**

This paper aimed to describe the development of the Living Donation Storytelling Project, a web-based digital library of living donation narratives from multiple audiences using video capture technology. Specifically, we aimed to describe the theoretical foundation and development of the library, a protocol to capture diverse storytellers, the characteristics and experiences of participating storytellers, and the frequency with which any ethical concerns about the content being shared emerged.

**Methods:**

This study invited kidney transplant recipients who had received LDKTs, living donors, family members, and patients seeking LDKTs to record personal stories using video capture technology by answering a series of guided prompts on their computer or smartphone and answering questions about their filming experience. The digital software automatically spliced responses to open-ended prompts, creating a seamless story available for uploading to a web-based library and posting to social media. Each story was reviewed by a transplant professional for the disclosure of protected health information (PHI), pressuring others to donate, and medical inaccuracies. Disclosures were edited.

**Results:**

This study recruited diverse storytellers through social media, support groups, churches, and transplant programs. Of the 137 storytellers who completed the postsurvey, 105/137 (76.6%) were white and 99/137 (72.2%) were female. They spent 62.5 min, on average, recording their story, with a final median story length of 10 min (00:46 seconds to 32:16 min). A total of 94.8% (130/137) of storytellers were motivated by a desire to educate the public; 78.1% (107/137) were motivated to help more people become living donors; and 75.9% (104/137) were motivated to dispel myths. The ease of using the technology and telling their story varied, with the fear of being on film, emotional difficulty talking about their experiences, and some technological barriers being reported. PHI, most commonly surnames and transplant center names, was present in 62.9% (85/135) of stories and was edited out.

**Conclusions:**

With appropriate sensitivity to ensure diverse recruitment, ethical review of content, and support for storytellers, web-based storytelling platforms may be a cost-effective and convenient way to further engage patients and increase the curiosity of the public in learning more about the possibility of becoming living donors.

## Introduction

Presently, over 740,000 people in the United States are living without functioning kidneys due to end-stage kidney disease (ESKD) [1,2]. In general, patients who can receive a living donor kidney transplant (LDKT) from a family member or friend live longer than those remaining on dialysis or waiting for years for a kidney from someone who has died [1,3]. The demand for kidneys continues to outweigh the supply; while nearly 100,000 patients are currently waiting for a kidney on the national transplant waiting list [4], LDKT rates have declined by 12% over the last decade, generally not exceeding 6500 kidneys annually [1]. There are also significant ethnic/racial disparities in LDKT [1,5-8]; in the last 15 years, Latinx, black, and Asian patients have become even less likely to get an LDKT compared with white patients than they were in the past [5], and they are also less likely to be donors [1].

Although increasing deceased donation rates is limited by practical and medical circumstances surrounding how individuals die, living donation rates are limited only by the number of healthy individuals who become aware, educated, and interested in donating 1 kidney while they are alive. Of the roughly 250 million adults in the United States, only 100,000 more individuals (0.04%) would need to agree to donate 1 kidney to eliminate the entire kidney donor shortage. Education strategies to increase LDKT commonly target patients and families using face-to-face educational sessions [9-14], home-based educational interventions [9,15-17], culturally targeted videos and websites in multiple languages, and decision-making aids [7,18-20]. Although effective, these interventions fail to reach (1) the general public [21,22]; (2) family members and friends who do not come to a transplant center to learn [23]; (3) kidney patients who are scared to ask others to donate on their behalf [19]; and (4) members of specific ethnic/minority groups who have cultural sensitivities to living donation [24], low health literacy [25], or greater medical mistrust of the health care establishment that are unaddressed through general education [26].

To expand the living donor pool, we need to reach beyond the walls of the transplant center to help patients share their interest in LDKT with more individuals and inspire more people who are still unaware of living donation to consider becoming donors. As few people know a living donor personally, we also need to help the general public realize that other people who look like them donate kidneys each year. Innovative strategies to educate and inspire more patients and potential living donors to consider living donation are needed.

Storytelling, the sharing of first-person narratives based on lived experience, is an educational approach that is authentic, emotional, and provides people with the opportunity to learn from others who look like them. Stories have the power to emotionally engage listeners, reach low-literacy audiences, and present complex information in informal and comprehensible ways [25,27-29]. Having opportunities for patients and living donors to share experiences and wisdom with each other is also an important tenet for excellence in patient-centered care [30]. Interventions that use storytelling as a means to produce behavioral change have been successful in increasing cancer screening rates [31], improving adherence to diabetes management behaviors [32], smoking cessation [33], reducing blood pressure [34], and losing weight [35]. Storytelling using digital apps also has the potential to reduce the burden of educating patients placed on providers [25]. As capturing stories using video software can be both expensive and complex, storytelling has only been used minimally, predominately in online communities and discussion forums [36,37] or health testimonials [38].

Developing ways to easily standardize and disseminate diverse living donor stories using digital technology could inspire more people to consider becoming living donors and reduce the kidney shortage nationally. This study aimed to describe the development of the Living Donation Storytelling Project, a web-based digital library of living donation narratives from multiple storyteller types (eg, recipient, donor). There were 4 aims: to describe (1) the theoretical foundation and development of a web-based digital library using video capture technology, (2) a recruitment protocol to capture diverse storytellers, (3) the characteristics and experiences of the participating storytellers, and (4) the frequency with which ethical concerns in the content shared emerged.

## Methods

### Theoretical Frameworks and Web-Based Library Development

The Narrative Theory supports the use of storytelling as an organic way in which humans naturally process and assign value to information, especially when it is presented by someone who resembles the listener [39-42]. The development of this web-based video library required much more than asking individuals to use their smartphones to film and upload stories. The formal phases of its development included selecting an audience of learners; using theory to determine the best delivery modality for that audience and the features it should have; drafting educational prompts to elicit high educational value; recruiting diverse storytellers to represent the entire transplant and living donation community; screening and editing stories to protect storytellers and eliminate sharing of misinformation; and building a web-based, searchable library of stories and marketing its availability to multiple communities (Multimedia Appendix 1).

On the basis of diffusion of innovation (DOI) theory [43], we chose a platform for recording and sharing stories that allowed storytellers to select a set of topics that they wanted to share about, then introduced each topic with an open-ended prompt, one at a time, to help them share it easily and clearly. This format satisfied DOI constructs of compatibility, trialability, observability, and relative advantage. The resource was highly compatible—consistent with cultural values and practices—as most people, even those facing socioeconomic challenges, have access to a smartphone [44-46] and are familiar with YouTube-style videos [47]. Our resource was trialable—users could try it out without committing to sharing a final video. After each prompt, the storyteller was able to review what they filmed, re-record it if necessary, and approve that footage before continuing. Observability—the ability to see the product in practice—was addressed with a sample video modeling how digital technology was used and by inviting potential storytellers to view completed stories. This provided a great relative advantage—ease of use and improvement over existing options—compared with having to record and edit your own video as the digital software automatically spliced together responses, creating a seamless story. Only when the storyteller was comfortable with the entire video, was it released for inclusion into the library.

As recommended by the transtheoretical model of behavioral change [48-50], when creating prompts for patients to share about, we considered that stories, especially those with higher emotional valence, have been shown to connect better with audiences who are earlier in their readiness to learn or take action. Therefore, we included prompts to probe for storytellers’ best moments after donation and recommendations for people facing similar situations to elicit sharing of more emotional content (eg, “When we learned that the surgery was a success, we all felt...” and “The best advice I could give someone else who is considering being a Living Donor is...”). We also included questions for learners who knew little about transplant and donation to invite them to learn more (eg, “I first considered donating a kidney...”). Storytellers were prompted to talk about the emotional and logistical challenges they faced during the LDKT process and how they overcame them. By doing so, they provided a road map for how the audience, if interested, could follow a similar path.

Social cognitive theory shows that people learn by observing others who look like them and by observing the consequences that others receive as a result of their actions [51,52]. Thus, we recruited real recipients, donors, and allies of many different backgrounds to boost identification with the storytellers. In addition, we built a filtering search function so that users of the library could search for storytellers who matched them in gender or race/ethnicity or were facing a similar situation (eg, needing a kidney, considering living donation; Multimedia Appendix 2). Finally, we developed prompts to enhance sharing in a way that allowed the listener to learn about the consequences of different decisions (eg, “Initially my attitude toward living kidney donation was...”; “Once I learned more about living donation, I considered becoming a living donor because...”; “I had questions and fears of my own, like... I was able resolve my concerns by...”).

The resulting Living Donation Storytelling Project web-based digital library [53] includes 5 key features to break open learning and pursuit of living donation in new ways: (1) digital video capture technology to film and seam videos together remotely without the need for an editor; (2) guided prompts to help storytellers select and share about topics most important to them using a mobile phone, tablet, or laptop; (3) a search engine to allow for audiences to locate stories most like them by demographic and story type; (4) the ability to upload content both to the library and social media to help individuals find living donors; and (5) referrals to additional educational content about LDKT, including the location of the nearest transplant center. The library is categorized into donor stories, recipient stories, family/friend stories, and stories from people in need of a living kidney donor. Although the website was launched only after a diverse representation of storytellers could be shown, the library of stories continues to widen as more people film and upload stories. Finally, Google Analytics metrics were enabled to track the usage of the site and its features, as well as views of specific stories.

### Storyteller Recruitment and Filming

**Figure 1:**
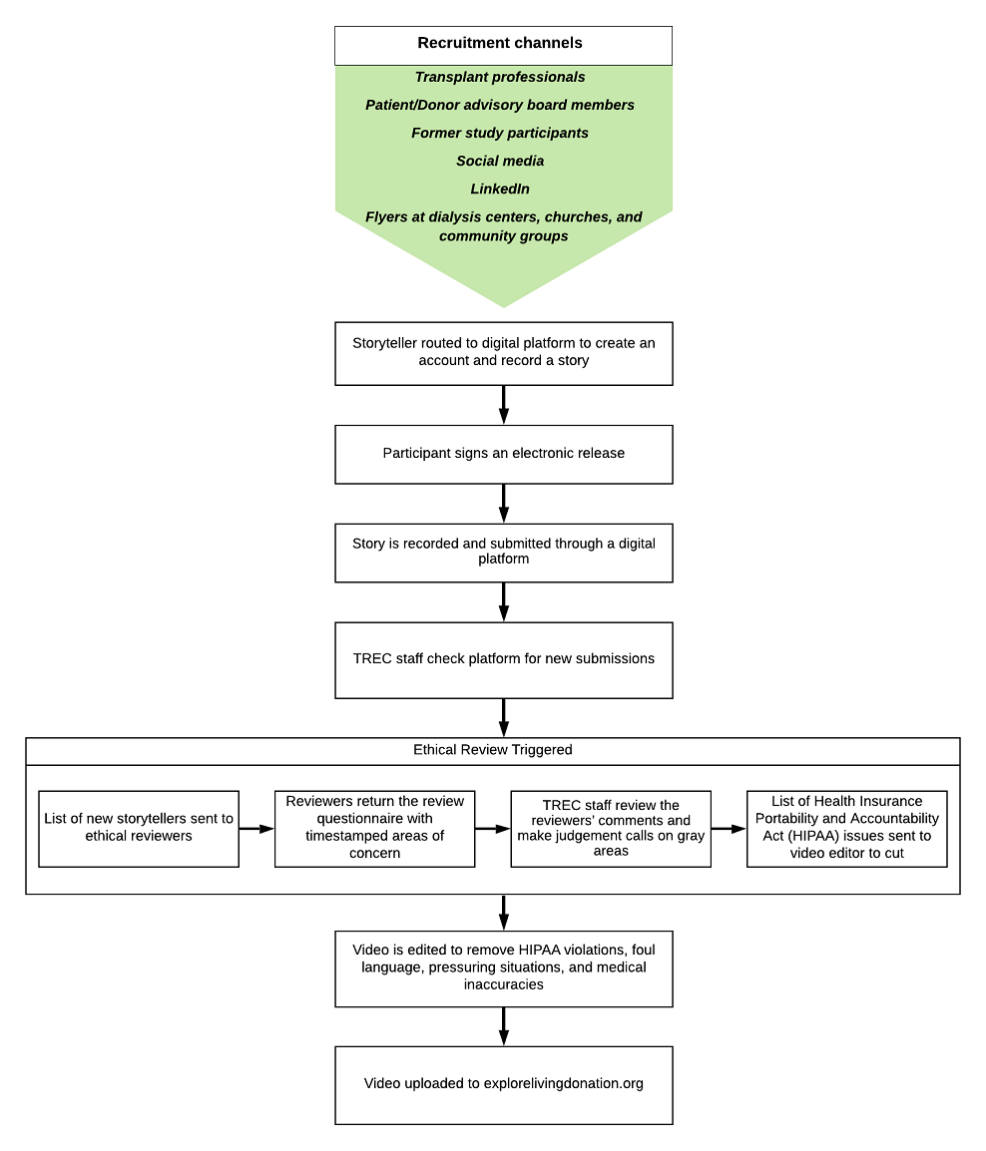
Process flow of initial recruitment of storyteller to a completed story by the Transplant Research & Education Center.

Storytellers were individuals who had previously donated a kidney, recipients of LDKTs, family members or friends, or kidney patients seeking an LDKT. Storytellers were recruited via social media (Facebook, Instagram, Twitter, and LinkedIn), community outreach at support groups and churches, and referrals from kidney professionals and organizations (Figure 1). Once a storyteller was identified, they were given a personal link to privately film their story using a story guide matching their storyteller type.

Prompts were developed by our research team and grouped as story guides based on 6 transplant story types: recipient, donor, family/friend, exploring donation, in need of a kidney, and kidney ally. Depending on the guide, storytellers were offered 5 to 25 open-ended prompts that addressed their decision-making experiences, questions, needs, fears, and hopes, their donation and transplant experiences, how their lives changed after donation or transplant, and recommendations for potential donors.

Storytellers completed a standard media release for use of the stories, received reminders not to disclose any protected health information (PHI), and were supported with questions by a staff member (Multimedia Appendix 3). Storytellers could also use a tool on the website to find their nearest transplant center (Multimedia Appendix 4). Once recorded, the video files were stored on the private servers of the video capture platform. The project underwent the University of California, Los Angeles (UCLA), Institutional Review Board (IRB) review (IRB#18-000516), where exempt status was awarded.

### Ethical Review of Story Content and Video Edits

After videos were submitted, transplant professionals (LH, SM, MA, and WB) watched each video and completed an ethical screening worksheet to check for PHI disclosures, about either the storyteller or anyone else, including their last name, addresses, transplant center name, social security numbers or medical record numbers, transplant date, or ESKD diagnosis date. They also screened for medical overgeneralizations or inaccuracies, pressuring language about donation or transplant, or foul language. Any problematic instances were coded by a timestamp and later removed by a video editor. The process flow of the storyteller’s path from the initial recruitment to the completed story is outlined in Figure 1.

### Storyteller Postsurvey

An opportunity to complete a 32-question postsurvey assessing the storyteller’s experience filming their story was offered to those who had already completed and uploaded their stories. Those who completed a story received a voluntary postsurvey link in the email address that they provided. Surveys were collected using the Research Electronic Data Capture (RED Cap) software, with each storyteller receiving a US $25 gift card after completing their survey. Data were stored on a secure, password-protected UCLA server.

The survey assessed storytellers’ demographic characteristics (eg, gender, race/ethnicity, and age), level of education obtained, and type of story completed (eg, donor, recipient, family member, etc). Storytellers were asked what motivated them to share their stories (eg, to dispel myths, to help more people become living donors). On a scale from 1-very difficult to 7-very easy, storytellers were asked how easy or difficult filming and sharing their story was.

## Results

### Diverse Storyteller Recruitment: Characteristics and Motivation

In total, 412 potential storytellers received an initial introductory email with a story link that was unique to their experience. Of those invited to participate, 34.7% (143/412) storytellers completed stories and, of these, 95.8% (137/143) completed a voluntary postsurvey. Among those who completed a story, 72.2% (99/137) of the storytellers were female, 76.6% (105/137) were white, 60.5% (83/137) were living kidney donors, and 81.0% (111/137) had a college degree or higher. About 8.8% (12/137) of the storytellers were Hispanic and 23.4% (32/137) were nonwhite (Table 1).

Nearly all (130/137, 94.9%) storytellers were motivated by a desire to educate the public about living donation (Table 2).

**Table 1:** Storyteller characteristics (N=137).

Characteristic		Values
Age (years), mean (SD)		49.6 (12.4)
**Gender, n (%)**		
	Male	38 (27.7)
	Female	99 (72.3)
**Race, n (%)**		
	White	105 (76.6)
	Black	14 (10.3)
	Other	18 (13.1)
**Ethnicity, n (%)**		
	Hispanic	12 (8.7)
**Story type, n (%)**		
	Living kidney donor	83 (60.6)
	Kidney recipient	37 (27.0)
	Family or friend of kidney recipient	7 (5.1)
	Patient on waitlist	3 (2.2)
	Family/friend of the patient on the waitlist	2 (1.5)
	Other	5 (3.6)
**Education, n (%)**		
	High school diploma or GED^a^	6 (4.4)
	Some college or vocational school	19 (14.0)
	College or vocational school degree	60 (44.1)
	Some professional or graduate school	11 (8.1)
	Professional or graduate school degree	40 (29.4)

^a^GED: General Education Development or General Education Diploma.

**Table 2:** Storyteller motivations, barriers, and disclosure of protected health information (N=137).

Responses			Value
**Storyteller motivations, n (%)**			
	To educate the public about living donation		130 (94.9)
	To spread awareness and help others		110 (80.3)
	To make a difference in living donor recipient’s and donor’s lives		109 (79.6)
	To help more people become living donors		107 (78.1)
	To dispel myths about living donation		104 (75.9)
**User experience, n (%)**			
	Storytellers who found filming a story difficult		34 (24.8)
	Storytellers who found filming their story emotionally difficult		29 (21.2)
**Disclosure of PHI^a^, n (%)^b^**			
	**PHI about themselves**		78 (57.8)
		Last name	55 (40.7)
		Specific transplant center	24 (17.8)
		Transplant or donation date, month, and year	25 (18.5)
		Geographic specifics of location	11 (8.1)
	**PHI about another**		56 (41.5)
		Last name	30 (22.2)
		Specific transplant center	15 (11.1)
		Transplant or donation date, month, and year	13 (9.6)
		Geographic specifics of location	4 (2.9)

^a^PHI: protected health information.

^b^Disclosure of PHI obtained from Ethical Review of Storytellers who completed the storyteller postsurvey (n=135).

### Storytelling Experience and Content Shared

On average, storytellers took approximately one hour to review the prompts and prepare and record a story using open-ended prompts within the video capture technology (mean 62.5; SD: 87.8 mins; range 5 mins-12 hours). Completed stories had an average length of 10 min (SD: 6:12 min; range 0:46 seconds - 32 min).

A quarter (34/137, 24.8%) of the storytellers stated that they had difficulty filming their story using the technology and 21.2% (29/137) of the participants found sharing their stories to be emotionally difficult (Table 2). Some reported difficulty navigating the video capture technology, whereas others lacked access to a smartphone, laptop, tablet, or any other device with a camera to record their story. Emotional barriers to sharing their stories included fear of being on film, fear of talking openly about needing a kidney transplant, and vulnerability associated with sharing their donation or transplant experience in a public forum. Although not statistically significant, older storytellers (>50 years of age) had more emotional difficulty sharing their story (37/137, 27.0% vs 20/137, 14.6%; *P*=.12) and filming their story (40/137, 29.2% vs 29/137, 21.2%; *P*=.37) using the technology than the younger storytellers.

Most storytellers answered the majority of the prompts that were available in their story guide. Tables 3 and 4 provide examples of what was shared for the most common prompts selected within various story guides. Recipients shared about how they coped with uncertainties about donation or transplant outcomes, including discussions of their faith, the support they received from their care team, and sources of information that they used. Recipients talked about renewed freedom—the ability to eat and drink what they wanted, travel, swim, and spend time with family. Donors talked about watching their recipient return to improved health, what motivated them to consider LDKT, fears they had for a loved one, fears they had for themselves, and how they resolved these fears, mostly through learning more. Those in need of a kidney, and their supporters, talked about how deserving they were and ways to get in touch with them if interested in being a donor.

Storytellers shared vulnerably about their experiences, both laughing and crying within their stories. Emotionally, people expressed gratitude for the gift of life, worry about whether the kidney would work, and concern for the health of the donor within their stories.

**Table 3:** Content shared by donors, recipients, and family around open-ended prompts.

Prompts by story guide		Examples of storytelling content shared
**Living donor**		
	The best advice I could give someone else who is thinking about being a living donor is...	“...just go ahead and ask questions. Talk to the transplant team. Let them decide, let the coordinators decide, do their screenings, do their questions. See if you are a likely candidate. You never know. I donated, maybe you can too.” (Lisa H)
	I ultimately decided to donate a kidney because	“Of course, there was nothing I wouldn't do to save my daughter's life. But, also, because the quicker she got off the [wait] list, the quicker someone else would get an opportunity.” (Luther)
	The best moment after my surgery was...	“About 4 days later, when I was visiting Lexi again. I saw how much happier and healthier she was [...] just seeing how good she was feeling really made me feel great.” (Luther)
**Kidney recipient**		
	My kidney failure began when I was (X) years old. At that time, I was doing (common activities for you before the transplant)...but then I started to notice (changes that affected your daily life)	“Kidney failure began when I was 25 years old, I was at stage 3. My kidney function was at about 40%. When I turned 31, and I became pregnant, that is when I become stage 5. My kidney function went from 40% functioning to about 8% functioning.” (Kara)
	Living without working kidneys meant that... The first time I had a dialysis treatment was... (explain how it felt)	“It meant that my time was limited. My disease started when I was 27, when I was 45, my kidney function has dropped to 9%. I felt defeated. A lot of the people that I talked to on my first day on dialysis had been coming there for 5 years.” (Rochelle)
	I found it... (difficult /easy) to talk about living donation with my family and friends, because...	“Found it easy to talk to about living donation with my friends and family. Because of living donation, I am alive today. So, I talk about it openly.” (Holly)
**Family member**		
	We learned as a family what kidney failure meant physically for (Recipient). However, for our family it also... (Explain the ways it changed your family’s life)...	“…For our family it meant a huge lifestyle change. It was a huge financial burden for our family. It was mentally draining for my parents especially. I was 15 at the time, so, I didn't really grasp Hans's situation.” (Drea)
	Some of the best resources I used to learn about kidney disease were...	“Living through Hans's situation as a family member. I was 14-15 when his life on dialysis began. It was an in-person, real life, first-hand experience.” (Drea)
	(Recipient's) dialysis schedule meant that we had to change our current plans because...	“Changed our family's plans for traveling. We like to travel a lot. It changed drastically. We hated going and having him left out. So, we worked around it and there were days that he didn't feel so great. So, dialysis sucks. Period.” (Annamarie)

**Table 4:** Content shared by potential donors and kidney patients around open-ended prompts.

Prompts by story guide		Examples of storytelling content shared
**Exploring donation**		
	I first considered donating a kidney...	“When our family found out that my niece Marie was in kidney failure. She was fairly young, she was about 13-14. She was about to start junior high and she was about start dialysis. That is when I first started to think about being a donor.” (Monica)
	Initially my attitude toward living kidney donation was...	“I was little, I was a afraid at first. I didn't know what it entailed and until I read about it more. I have two kidneys and I have one to spare.” (Kurt)
	Once I learned more about living donation, I considered becoming a living donor because...	“Once I learned about kidney donation, I considered to be a donor because my wife had kidney failure.” (Kurt)
**In need of a kidney**		
	I could get a kidney from a living donor. So far...(tell what you have done or plan to do to find a donor)	“It is hard for me to find a kidney because; I need a B-, live kidney donor. I’ve done a lot of social outreach. I've made shirts, made some pamphlets, and gone on social media [to find a donor].” (Kabir)
	I started having these symptoms... I was diagnosed with (explain prognosis), (X) months/years ago	“I was having high blood pressure and slowly I was feeling fatigue and I would always have this headache. I was not aware of what was going on with my health. I thought I was just too active. But, slowly the disease took over me and I had stage 4 kidney failure.” (Kabir)
	To stay alive, I have to go for dialysis, which is... (briefly describe what it is, how often you go, your new quality of life)	“For my dialysis, peritoneal dialysis every day. Most people do couple of hours every day but I do about 12 and a half hours every day [...]” (Kabir)

### Ethical Concerns: Disclosure of Protected Health Information or Inaccurate Information

More than half (78/135, 57.8%) of the storytellers disclosed PHI about themselves, most often their last name, specific transplant center name, transplant or donation date, and geographic details about their locations. Some storytellers, 41.5% (56/135), also shared PHI about others involved in their living donation experience (Table 2).

A minority (18/135, 13.3%) of the storytellers shared medical inaccuracies or overgeneralizations. Examples included, “…going on dialysis means your life is over and your caregivers’ life is over” or “…1 in 3 actually have kidney disease.” Statements that could be considered instances of pressuring were shared by 11.9% (16/135) of the participants. These included statements like “People who do not give to a family member are selfish.”

## Discussion

### Principal Findings

This study evaluated the feasibility of building a web-based digital library and recruiting storytellers to share their experiences. Using community-based participatory research practices and recruitment through social media, we successfully recruited storytellers involved with living donation who were predominately female, white, and motivated to assist others in making choices about donation and transplant. In general, it was more difficult to recruit minority storytellers and males to share their stories. As African Americans and Hispanics have 2.9 and 1.3 times higher ESKD incidence rates, respectively, compared with their white counterparts [1] and the lifetime risk of ESKD is higher in males than in females [1], additional research is needed to explore how to enroll these communities into sharing their stories and help them feel supported when sharing their stories.

Thousands of kidney patients awaiting transplant die each year before a matching kidney is found [1]. The Living Donation Storytelling Project is an important public resource enabling real-life stories about living kidney donation from multiple audiences to be captured using video capture technology and shared through a web-based searchable digital library.

At the start of this project, we were unclear how difficult storytelling would be and what types of content would be shared. More than half of those who were offered a link to record a story did not submit a completed video story, and about one-quarter of participants who completed a story reported either technical or emotional challenges when asked to reflect afterward on the recording process. However, the content shared, including poignant first-person recounting of fears, lessons learned, challenges overcome, and recommendations for others facing these decisions were very powerful. Future research should determine the key topics most commonly shared by storytellers and assess which storytellers and types of content most connect with different audiences. Further examination of the impact of storytelling combined with other, more traditional, educational strategies for increasing the number of living donors coming forward and LDKT rates is also needed.

Transplant professionals who served on our ethical review board concluded that, in general, storytellers act ethically; however, reminders should be sent to ensure privacy and prevent the disclosure of PHI. Clarification of what not to share in a public forum helped reduce disclosure of PHI, as did editing afterward. Additional work is still needed to explore ethical issues, including whether storytellers seeking living donors should be allowed to disclose their contact information in videos shared in the library.

Moving forward, there are many applications of the Living Donation Storytelling Library methodology. Stories can be easily incorporated within the traditional educational process during transplant or donation evaluation, provided as a general introduction to transplant in dialysis centers, or embedded into educational portals linked to electronic patient medical records. Education delivered through these portals is quickly becoming a part of the standard of care [54-57]. As social media has become a common forum for prospective recipients and donors to seek donors, this library also provides interested patients with an easy way to share their interest in finding a living donor widely with their social media communities [22,58,59]. This library can also be used to amplify the voice of transplant champions and ambassadors to increase public awareness about the cause [60]. Sharing stories through social media may also be an effective way to advance a potential living donor’s or transplant patient’s stage of readiness for LDKT [61].

Finally, the Living Donation Storytelling Project may be particularly effective for reaching certain groups who are not well-served by existing educational strategies. Specifically, stories may also be a gentler way to introduce the option of living donation for patients who are concerned about harming a loved one, those who cannot read or have difficulty reading [62], those who speak languages other than English [25], and those with higher levels of medical mistrust [63-66]. Stories may also be well-suited for educating patient populations, including Native Americans or First Nations people who have cultures steeped in oral traditions [67-70]. Finally, the digital storytelling library methodology can be applied to health areas outside of living donation, as first-person storytelling has been shown to improve health outcomes for patients who belong to racial or ethnic minorities, have low health literacy, and are of lower socioeconomic status [71].

### Conclusions

In summary, watching real-life stories can be reassuring, empowering, and, sometimes, inspiring [25,31,72,73]. With appropriate sensitivity to ensure diverse recruitment, ethical review of content, and support for storytellers while using innovative technology, digital storytelling technologies may be a cost-effective way to further engage patients and increase the curiosity of the public about becoming living donors.

## Appendix

Multimedia Appendix 1 Steps for building a digital library.

Multimedia Appendix 2 Living Donation Storytelling Project website search engine.

Multimedia Appendix 3 Living Donation Storytelling Project storytelling instructions.

Multimedia Appendix 4 Living Donation Storytelling Project transplant center search tool.

## Abbreviations

DOI: diffusion of innovation
ESKD: end-stage kidney disease
IRB: Institutional Review Board
LDKT: living donor kidney transplant
PHI: protected health information
UCLA: University of California, Los Angeles
