# Supplementary material for: A Digital Library for Increasing Awareness About Living Donor Kidney Transplants: Formative Study
Source: JMIR Form Res. 2020 Jul 21;4(7):e17441. doi: 10.2196/17441 (PMC7404010; doi:10.2196/17441)
Supplement: Multimedia Appendix 1 [file formative_v4i7e17441_app1.pdf]

STEPS FOR BUILDING A DIGITAL LIBRARY

1 SELECT AN AUDIENCE

Select an audience of learners

2 DETERMINE THEORETICAL APPROACH & MODALITY

Use behavioral theories to determine the best delivery modality for the target audience and necessary features

3 DRAFT PROMPTS

Draft prompts to probe for valuable change-talk and other educational content that can be used to inspire change in knowledge, attitudes, readiness, or behavior

4 RECRUIT DIVERSE STORYTELLERS

Recruit diverse storytellers who are representative of the larger target community

5 SCREEN AND EDIT STORIES

Ethically review and edit stories to protect storytellers and eliminate sharing of misinformation

6 BUILD AN ONLINE, SEARCHABLE LIBRARY

Conduct web development to ensure that the resource is searchable and easy to use

7 MARKET THE RESOURCE

Invite multiple communities to participate in using the new resource
