# Supplementary material for: A Digital Library for Increasing Awareness About Living Donor Kidney Transplants: Formative Study
Source: JMIR Form Res. 2020 Jul 21;4(7):e17441. doi: 10.2196/17441 (PMC7404010; doi:10.2196/17441)

EXPLORE Living Donation

Home | Stories | Learn | About

All Videos

Search and browse all the videos

This library keeps growing. Watch the videos we have now and please consider adding your own story.

Select a gender

Select a location

Select a race / ethnicity

Select a living donation story type

See results

Search the library | Tell your story | Learn more | Subscribe for updates

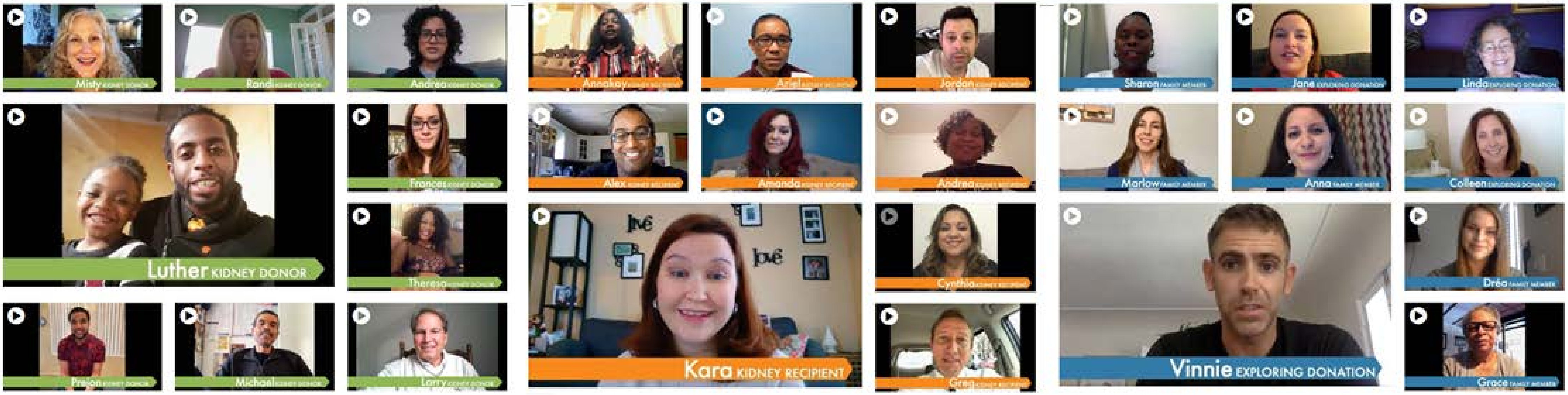

Supplement: Multimedia Appendix 2 [file formative_v4i7e17441_app2.pdf]
