# Supplementary material for: A Digital Library for Increasing Awareness About Living Donor Kidney Transplants: Formative Study
Source: JMIR Form Res. 2020 Jul 21;4(7):e17441. doi: 10.2196/17441 (PMC7404010; doi:10.2196/17441)
Supplement: Multimedia Appendix 3 [file formative_v4i7e17441_app3.pdf]

Want some tips on using the digital platform and filming your story?

Watch this video and download the pdfs below.

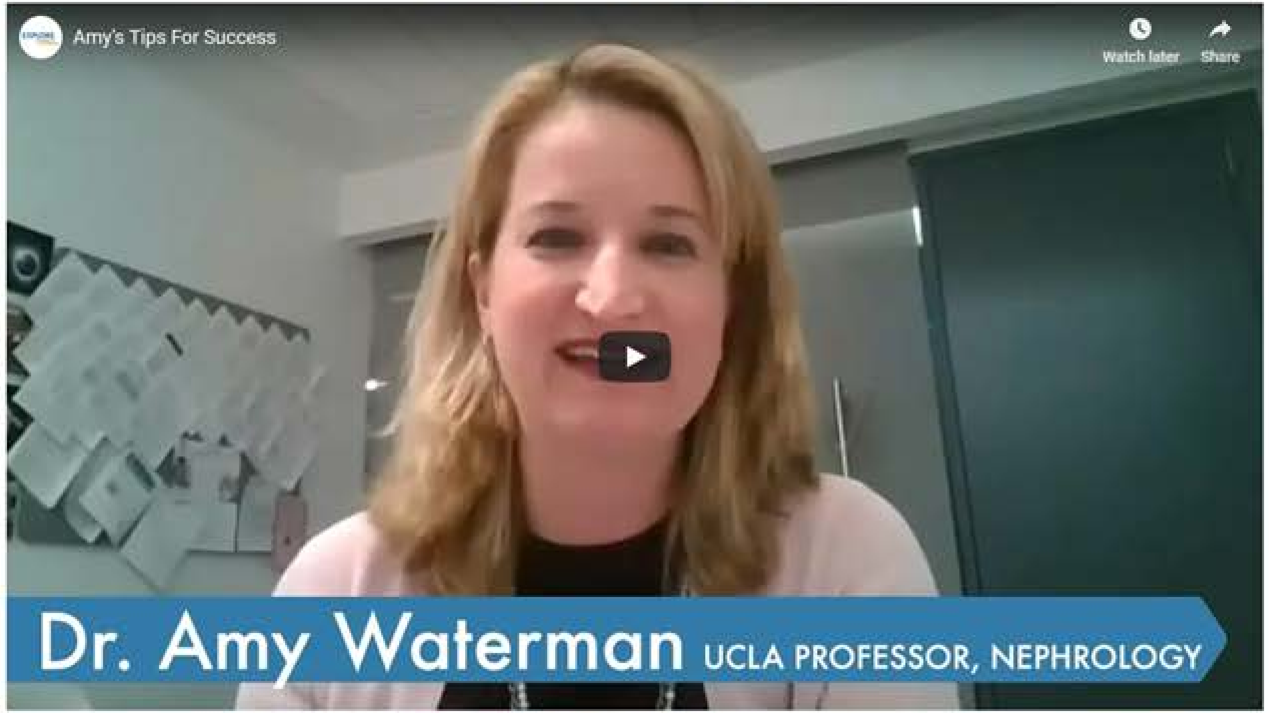

How to submit videos (pdf)

Filming instructions & tips (pdf)

TREC Transplant Research & Education Center

TERASAKI RESEARCH INSTITUTE

Filming Instructions & Tips:

IMPORTANT:

- **DO NOT state your last name, the full name of others involved with your story, or your transplant center.**
- **Please sit in front of a neutral background with no advertisements or marketing** information to keep the focus on you and your words.
- **Make sure you are not backlit.** This often happens if sitting with a window directly behind you.

1. **What to wear**
   a. Put on your favorite top and wear pajama bottoms if you want, viewers will only be able to see your torso. What's most important for this video is that you are comfortable.
2. **Light is key**
   a. Your light source should come from in from in front of you or from the side, not behind.
   b. More lighting is almost always better.
3. **Position of camera**
   a. Depending on which device you use to record (e.g., smart phone, laptop, tablet) you will need to position the device so that the webcam captures your face in a flattering way. Simple backgrounds are best so as not to distract the viewer from
