# Supplementary material for: A Digital Library for Increasing Awareness About Living Donor Kidney Transplants: Formative Study
Source: JMIR Form Res. 2020 Jul 21;4(7):e17441. doi: 10.2196/17441 (PMC7404010; doi:10.2196/17441)

Find a transplant center near you

Zip or city or state: 90024 | Search this radius: 10 mi | Results: 50 | Search

University of California at Los Angeles Medical Center
757 Westwood Plaza
Los Angeles CA 90095
United States

Phone: (800) 825-2631

0.6 mi
Directions

Cedars-Sinai Medical Center
8700 W. Beverly Boulevard
Los Angeles CA 90048
United States

Phone: (800) 303-6235

3.3 mi
Directions

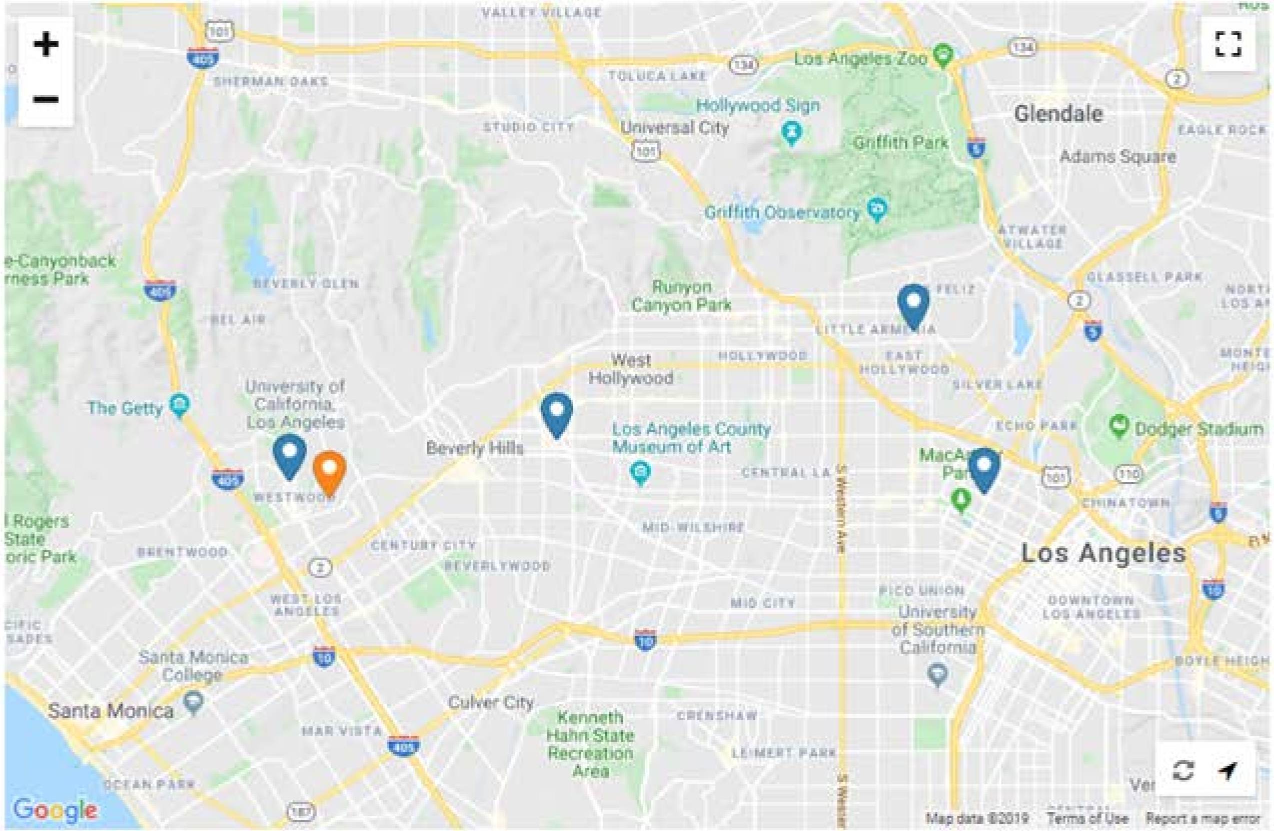

Supplement: Multimedia Appendix 4 [file formative_v4i7e17441_app4.pdf]
